# St. Bartholomew's Hospital and the Wounded

**Published:** 1914-11-21

**Authors:** 


### ST. BARTHOLOMEW'S HOSPITAL AND THE WOUNDED.

### Two Months' Experience of War Work.

On September 1 the Governors of the Hospital placed the whole of the east wing, containing 174 beds, at the disposal of the War Office, for wounded and sick soldiers During that month the transport of men from the Front home to England was a matter of considerable difficulty, and so, with the leave of the authorities, the upper two wards of the wing were used for men upon whom operations were performed to fit them for enlisting. Thus during the waiting time much good work was done. The wing is worked as a section of the 1st London (City of London) General Hospital, a base hospital, the staff of which is entirely drawn from the staff of St. Bartholemew's.

### Wounded Retreat before the German Advance.

On the evening of October 14, with only half an hour's notice, seventy-eight wounded Belgian soldiers were received. Without confusion, in spite of so short a warning, the whole of these were transferred from the motor-cars and ambulances in the space of thirty-five minutes by the help of members of the resident staff, dressers, and students, under the direction of Major A. E. Garrod, Major W. McAdam Eccles, and Captain Girling Ball. The men were extremely tired and hungry, but through the unflagging labours of the sisters and nurses they were soon in bed, fed, and their wounds attended to, when, without any delay, most were fast asleep, only too pleased to be out of the trenches and into comfortable quarters. The following day a further fifty-two Belgians were admitted. The majority of the men had been wounded three, or even four, weeks previously at Liége, Malines, or Louvain, while others much more recently, at Antwerp. They had been transferred from hospital to hospital as the Germans advanced towards the coast. For the most part the wounds were not severe, except for septic open fractures of the upper limb, and in one instance of the mandible. Only 10 per cent. were "sick," rheumatism and myalgia being prevalent complaints. All these cases of the Belgians have done well, a good many bullets, chiefly from shrapnel, and fragments of shell having been extracted. Most of the Mauser bullet wounds have been comparatively free from infection, but the shrapnel and shell wounds were often much infected, and have taken several weeks to heal. Among the Belgian cases there were none of tetanus or of emphysematous gangrene.

On October 24 Princess Clémentine of the Belgians, with her husband, Prince Victor Napoleon, visited the wing and spent some time in conversing with the wounded. On the following day the Duchesse de Vendôme and the Duc de Vendôme honoured the hospital with their presence, and the men greatly appreciated the sympathy they showed. The majority of the Belgians have now left the hospital for convalescent homes in various parts of the country—homes provided by most generous persons for this purpose. Many have since returned to the fighting-line, where they are once more showing that bravery and courage which have so characterised the soldiers of Belgium.

### Mauser Bullet Wounds

As the wards have been emptied of Belgians, British soldiers, wounded and sick, have been received up to the number of 209. The wounds of these have been much more recent, and many of them a good deal more severe than those of our Belgian allies.

The majority of the bullet wounds, both Mauser and shrapnel, have had entrance and exit apertures, and many of them have been associated with grave injury to bone, joint, and nerve. The Mauser bullet, if it does not touch bone, makes a very clean wound, and rarely remains in the tissues. When it does so stay, it not infrequently turns round, so that, when discovered by *x*-rays, its sharp end is found pointing towards the wound of entrance, which wound may be as small as or smaller than a threepenny-bit. The proportion of wounds of the upper and lower extremities to wounds of the trunk is very great, and wounds of the forearm and of the foot are very common.

### Two Interesting Cases.

Naturally, severe wounds of the abdomen and thorax do not find their way often into English hospitals; but some where the bullet has traversed the chest or abdominal cavity without any permanent damage have already been seen at St. Bartholomew's. In one remarkable case a Mauser bullet was found by a stereoscopic skiagram to be lodged in the hilum of the right lung, and yet the man had no untoward symptom and has left the hospital retaining his trophy!

Another very interesting case was that in which a piece of shrapnel casing was discovered by a skiagram to be in the muscles at the back of the thigh, while the entrance wound was over Hunter's canal. The wound was enlarged, the femoral artery exposed, and it was found that the fragment of metal had passed right through the vessel and into the tissues behind it. The clot which was controlling the exit of blood from the artery was accidentally displaced, furious hæmorrhage temporarily occurred, and the artery had to be ligatured on the proximal and distal side of the jagged wound in its wall. After this the fragment of shrapnel was removed, and the man has done well. One or two slight cases of secondary bleeding have been seen, but the hæmorrhage was easily controlled. Only one case of tetanus, up to November 14, had occurred at St. Bartholomew's, and this was a very acute one, ending fatally within twenty-four hours, in spite of every appropriate treatment.

Their Majesties the King and Queen have paid a visit to the hospital, spending over an hour and a half there, and speaking to every man, both British and Belgian. Their cheering words were much appreciated, and brought comfort to all.

